# Single and composite damage mechanisms of soil polyethylene/polyvinyl chloride microplastics to the photosynthetic performance of soybean (*Glycine ma*x [L.] merr.)

**DOI:** 10.3389/fpls.2022.1100291

**Published:** 2023-01-18

**Authors:** Haibin Li, Fupeng Song, Xiliang Song, Kongming Zhu, Qun Lin, Jinliang Zhang, Guoqiang Ning

**Affiliations:** ^1^ Department of Soil Science, College of Resources and Environment, Shandong Agricultural University, Tai’an, China; ^2^ Dongying District, Agricultural and Rural Bureau, Dongying, China

**Keywords:** microplastics (MP), oxidative damage, photosynthesis, soybean, soil contamination

## Abstract

**Introduction:**

Adverse impacts of soil microplastics (MPs, diameter<5 mm) on vegetative growth and crop production have been widely reported, however, the single and composite damage mechanisms of polyethylene (PE) /polyvinyl chloride (PVC) microplastics (MPs) induced photosynthesis inhibition are still rarely known.

**Methods:**

In this study, two widely distributed MPs, PE and PVC, were added to soils at a dose of 7% (dry soil) to examine the single and composite effects of PE-MPs and PVC-MPs on the photosynthetic performance of soybean.

**Results:**

Results showed PE-MPs, PVC-MPs and the combination of these two contaminants increased malondialdehyde (MDA) content by 21.8-97.9%, while decreased net photosynthesis rate (Pn) by 11.5-22.4% compared to those in non-stressed plants, PVC MPs caused the most severe oxidative stress, while MPs stress resulted in Pn reduction caused by non-stomatal restriction. The reason for this is the single and composite MPs stress resulted in a 6% to 23% reduction in soybean PSII activity RCs reaction centers, along with negative effects on soybean PSII energy uptake, capture, transport, and dissipation. The presence of K-band and L-band also represents an imbalance in the number of electrons on the donor and acceptor side of PSII and a decrease in PSII energy transfer. Similarly, PVC single stress caused greater effects on soybean chloroplast PSII than PE single stress and combined stresses.

**Discussion:**

PE and PVC microplastic stress led to oxidative stress in soybean, which affected the structure and function of photosynthetic PSII in soybean, ultimately leading to a decrease in net photosynthetic rate in soybean.

## 1 Introduction

Plastics are widely used in people’s daily life due to their corrosion resistance, chemical stability, and convenient production (Shen et al., 2019). In 2021, global plastic production has exceeded 300 million tons, and more than 50% of which were single-use plastic products (Chen et al., 2021). In the process of plastic being consumed, MPs with particle size less than 5.0 mm will be generated due to mechanical friction, light, thermal radiation and biological manner (Bosker et al., 2019). The corrosion resistance of MPs makes them difficulty to degradation at natural environment conditions (Hernandez et al., 2017; Allouzi et al., 2021), and difficult to biodegrade (Aliyari Rad et al., 2022). The total amount of worldwide MPs is expected to reach 12 billion tons in the next 30 years, which will be distributed in various natural environments and life organisms (Ya et al., 2021). For example, MPs were found in varying concentrations in agricultural soils in Shanghai (mean 78.0 items kg^-1^) and Wuhan (mean 2020 items kg^-1^) of China, in industrial soils in Sydney (300∼67500 mg kg^-1^) of Australia, and in floodplain soils in Switzerland (593 items kg^-1^) (Fuller and Gautam, 2016; Liu et al., 2018; Chen et al., 2020). Up to now, most of the current studies focused on MPs in the marine ecosystem (Mofijur et al., 2021). While few studies have focused on the potential threats to terrestrial biota (Jambeck et al., 2015; Huang et al., 2020; Möller et al., 2020) although it is estimated more that 80% of MPs in the ocean are from terrestrial ecosystems.

Higher plants are an important part of terrestrial ecosystem (de Souza Machado et al., 2019; Yin et al., 2021). In recent years, the effects of MPs on plants have been well documented (2020; Kalčíková et al., 2017; Meng et al., 2021; Pignattelli et al., 2021; Rozman et al., 2021). It has been reported that the entrance of MPs into soil adversely affected a series of plant performance including produce excess reactive oxygen species (ROS) (Zong et al., 2021; Shahi Khalaf Ansar et al., 2022), block nutrients transport (Huang et al., 2019; Jiang et al., 2019), injure cell structure (Mao et al., 2018), alter metabolic systems (Wu et al., 2020), inhibit the synthesis of leaf pigments and photosynthetic activity (Wu et al., 2019), reduce plant biomass (Sorrentino et al., 2017; Qi et al., 2018), resulting in negative impacts on plant community structure and ecosystem functioning (Lozano et al., 2021). Among all of biological processes in plants, assimilation of carbon dioxide by photosynthesis is important for plants (Wen et al., 2011) due to its key role in organic substance synthesis and accumulation, nutrient uptake and transport, and changing environment sensitivity and adaptation (Kumari et al., 2020). The inhibitory effect of MPs on the photosynthetic performance have been detected in both of aquatic and terrestrial organisms. For instance, Sorrentino et al. (2017) demonstrated that polystyrene (PS) nanoplastics (50 mg L^-1^) negatively affected photosynthetic metabolism systems in leaves of cucumber, resulting in a remarkable biomass reduction. A recent study conducted by Teng et al. (2022) showed that soil PE MPs (1000 g kg^-1^) seriously suppressed gene expression in photosynthetic systems, inhibited chlorophyll synthesis and Rubisco activity, reduced net photosynthetic rate and respiration in the dark reaction phase, prevented electron transport, and resulted in a downregulation of photosynthesis in leaves of *Nicotiana tabacum*. Reduced photosynthesis affects plant carbon assimilation capacity and reduces plant stress tolerance (Khadem Moghadam et al., 2020). Also, polypropylene (PP) and PVC MPs at a high concentration inhibit algae photosynthetic activity by lowering the quantum yield or potential photosynthetic activity of PSII reaction centers (Wu et al., 2019). PE and PVC-MPs inhibited the growth and photosynthesis of several algae (Delangiz et al., 2022). The photosynthetic inhibition may be due to the direct toxicity of benzene deriving from MPs degradation (Li et al., 2020), resulting in ROS accumulation and lipid peroxidation (Gao et al., 2019), chlorophyll (Chl) biosynthesis inhibition, cell structure and photosynthetic system destruction (Mao et al., 2018), gene expression downregulation (Teng et al., 2022), key metabolisms disturbance (Zhou et al., 2021), nutrient cycling retard (Huang et al., 2019; Jiang et al., 2019), and light energy utilization efficiency reduction (Zhou et al., 2019).

Soybeans are an important oil-bearing crop, and soybean oil and its processing products play an important role in biofuels, edible oils and livestock feeds. The global soybean planted acreage in 2021 is 35.43 million hectares with a total production of 366 million tons. With 261.8 million tons of soybean production in 2017 66% of global soybean imports, China’s soybean production and security is an ongoing topic of debate (Liu et al., 2021). It’s estimated that 41% of soybean oil is used as biofuel in the U.S. in 2021, and 85.34% of soybeans are used for foods or biofuels in China (Guo et al., 2022). In addition, soybean has the advantages of short growth period and easy to culture, making it an excellent choice for studying microplastic pollution in terrestrial ecosystems (Kuligowski et al., 2022). Up to now, although noteworthy progresses have been made recently in elucidating the negative effects of MPs on plant photosynthesis, the effects of single type of MPs and the combined phytotoxicity of different types of MPs on plant photosynthetic function in leaves of soybean still unknown (de Souza Machado et al., 2019; Dong et al., 2020). In this study, two of most common thermoplastics, PE and PVC, were selected as the test MPs. We hypothesized that if soybean is exposed to MPs in field, the combination of PE-MPs and PVC-MPs will show higher toxicity on the photosynthetic performance of soybean than PE-MPs or PVC-MPs alone. The main objectives of the study were: (1) to ascertain whether adverse effects on photosynthesis of soybean would be induced by PE-MPs and PVC-MPs; (2) to distinguish the differences in the photosynthetic responses between PE-MPs, PVC-MPs, and PE+PVC-MPs; and (3) to elucidate the mechanisms by which MPs cause stress on plant photosynthetic performance. The results will be helpful in promoting the knowledge of phytotoxicity mechanisms of coexistence of PE-MPs and PVC-MPs in the photosynthetic performance of soybean and their potential risks on agricultural security.

## 2 Materials and methods

### 2.1 Experimental field and materials

The experimental field was located in the Experiment Station of Shandong Agricultural University (36°09’N, 117°09’N). The basic physical and chemical properties of experimental field soil (at a depth of 0-20 cm) were as follows: pH 7.89, bulk density 1.37 g cm^-3^, soil organic matter 18.13 g kg^-1^, total nitrogen 1.46 g kg^-1^, available phosphorus 17.99 mg kg^-1^, and available potassium 197.43 mg kg^-1^. The study site has a temperate continental subhumid monsoon climate. Its annual average temperature was 12.9°C, and annual accumulated precipitation was 750 mm, respectively.

Experimental PVC-MPs and PE-MPs were purchased from Huachuang Chemical Co., Ltd. (Guangdong, China). The two MPs were passed through a sieve to get a particle size of 13 μm. Seeds of soybean (*Glycine ma*x [L.] merr.) (c.v. Zhonghuang 37) were purchased from local market and disinfect with 10% H_2_O_2_ for 10 minutes, then rinsed well with distilled water.

### 2.2 Experiment design

A plot experiment was conducted with four treatments: control (CK), PE (addition of PE-MPs); PVC (addition of PVC-MPs); and PE+PVC (addition of PE-MPs and PVC-MPs). The dose of PE and PVC treatments were 7.0% and 7.0% ω/ω dry weight of surface soil (0-20cm depth), respectively. The PE+PVC treatment comprise of PE-MPs and PVC-MPs with the dose of 3.5% ω/ω dry weight of surface soil, respectively. Soil MPs contents were selected based on field investigations and literature review of MPs concentrations in soils (Ge et al., 2021). Each experimental treatment was repeated three times, there were 12 plots in total with a completely randomized design. The area of each plot was 9.0 m^2^ (3.0 m×3.0 m). The distance between different plots was 1.0 m. After different doses and types of MPs were manually mixed into the surface soil and balanced for one month, soybean seeds were sown in the soil with row spacing of 33 cm and seedling spacing of 27 cm on 20th April 2021. The soil moisture was maintained at 70–80% using an automatic drip irrigation system. The application rate of chemical fertilizer was based on local farmer’s conventional practice, compound fertilizer (N 15%-P_2_O_5_ 15%-K_2_O 15%) was applied into soil between soybean rows in strips at the soybean seedling stage with an amount of 600 kg hm^-2^ in each plot. All the parameters were measured at the flowering stage of soybean (75 days of cultivation, July 4, 2021), also one apical second leaf of soybean from each replicate of the four treatments was taken and frozen using liquid nitrogen and then used to determine malondialdehyde and T-SOD activity; mature soybeans were removed from the soil at 110 days (August 8, 2021) of soybean planting. All soybean assays are three replicates of each of the four treatments sampled once and assayed once.

### 2.3 Leaf chlorophyll content and gas exchange parameters

The second apical soybean leaves were selected to conduct the measurements. The chlorophyll (Chl) content (SPAD value) was measured using a chlorophyll meter (SPAD-502, Minolta, Japan). A portable open photosynthesis measurement system (CIRAS-3, PP-system, Hitchin, England) with a built-in blue-red light source was used to perform leaf gas exchange measurements between 9:00-11:30 A.M. on a sunny day. The light intensity, CO_2_ concentration and leaf temperature of CIRAS-3 were set as 1200 μmol m^-2^ s^-1^, 400 μmol mol^-1^, and 25°C, respectively (Teng et al., 2022).

### 2.4 Leaf OJIP transient

The leaf OJIP transient measurements were determined with dark-adapted soybean leaves by using a saturating pulse analysis method of Multi-Function Plant Efficiency Analyzer (M-PEA, Hansatech Ltd., Norfolk, England). A beam of saturating red-light pulse (5000 μmol m^-2^ s^-1^) was emitted to trigger the chlorophyll A fluorescence transient. The chlorophyll a fluorescence transients were analyzed with the JIP-test (Manaa et al., 2021). The Chl fluorescence parameters accompanied with formulas are listed in **Appendix (Table S1)**.

### 2.5 Leaf malondialdehyde (MDA) content and total superoxide dismutase (T-SOD) activity

MDA and T-SOD assay kits (Nanjing Jiancheng Institute of Biological Engineering, Nanjing, China) were used to measure the MDA content and T-SOD activity of soybean leaves according to the manufacturer’s instructions for use of the reagents. Soybean leaves were homogenized in an ice water bath and the homogenate was centrifuged at 5000 rpm for 15 minutes and the supernatant was taken. After the suspension was removed, normal saline was added into the residues to obtain the homogenates (10.0%, w/v). After the homogenates were centrifuged at 2,000 rpm min^-1^ at 4 °C for 15min, supernatant was obtained to conduct T-SOD analysis (hydroxylamine method) and MDA analysis (TBA method), and finally were measured by microplate system (Çatav et al., 2021; Zhang et al., 2021).

### 2.6 Soil analysis methods

Three undisturbed soils were collected using stainless steel rings (volume 100 ml) Before the start of the experiment for determination of soil bulk density (Tian et al., 2022). Subsequently, three pieces of soil topsoil (0-20 cm) were randomly collected in the field, naturally dried in a light-proof environment and then sieved and set aside. Ten grams of air-dried soil was added to a beaker containing 25 mL of deionized water. The mixture was stirred with a glass rod to fully disperse the soil particles and left to stand for 30 min. A pH meter was used to measure the pH of the supernatant (Dong et al., 2021). Ferrous sulfate titration was used to determine the soil organic matter content (Gallardo et al., 1987). The total nitrogen was determined by the Kjeldahl method (Sáez-Plaza et al., 2013). The available potassium with flame spectrophotometer after extracted using ammonium acetate (Zhang et al., 2022), and the available phosphorus concentration was measured by an acid-extracted molybdenum colorimetric method (Dong et al., 2021).

### 2.7 Data analysis

The data were statistically analyzed using SPSS 22.0 software (IBM, United States). Means and standard deviations (±SD) are shown in the graphs. Significant differences (*P* ≤ 0.05) between treatments were based on one-way ANOVA using Duncan’s test. Statistical plots in the text were produced using Origin 2019b (Origin Lab, United States).

## 3 Results

### 3.1 Soybean leaf MDA and Chl contents

**Figure 1:**
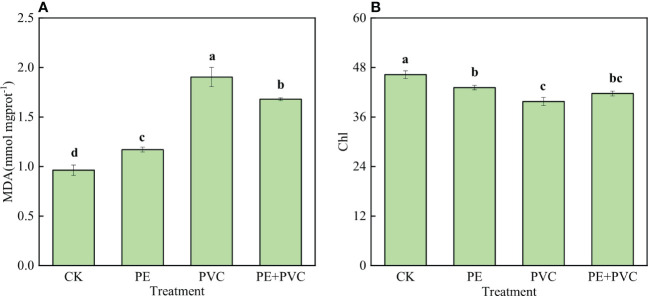
Leaf malondialdehyde (MDA) **(Figure-A)** and chlorophyll (Chl) **(Figure-B)** content of soybean leaves under MPS stress. Different lowercase letters in the graph represent significant differences between treatments (P≤0.05).

The MDA contents and SPAD values in leaves of soybean from different soil MPs treatments are presented in **Figure 1**. Different PE and PVC stresses significantly increase leaf MDA contents (**Figure 1A**). Compared to CK treatment, MDA contents in the PE, PVC and PE+PVC treatment enhanced by 21.9%, 97.9%, and 75.0%, respectively. The Chl contents were remarkable decreased in different soil MPs-treated soybeans. Compared with that of CK treatment, the soybean leaf SPAD values in the PE, PVC, and PE+PVC treatments decreased by 6.9%, 14.0%, and 9.9% respectively (**Figure 1B**).

### 3.2 Soybean leaf T-SOD activity

**Figure 2:**
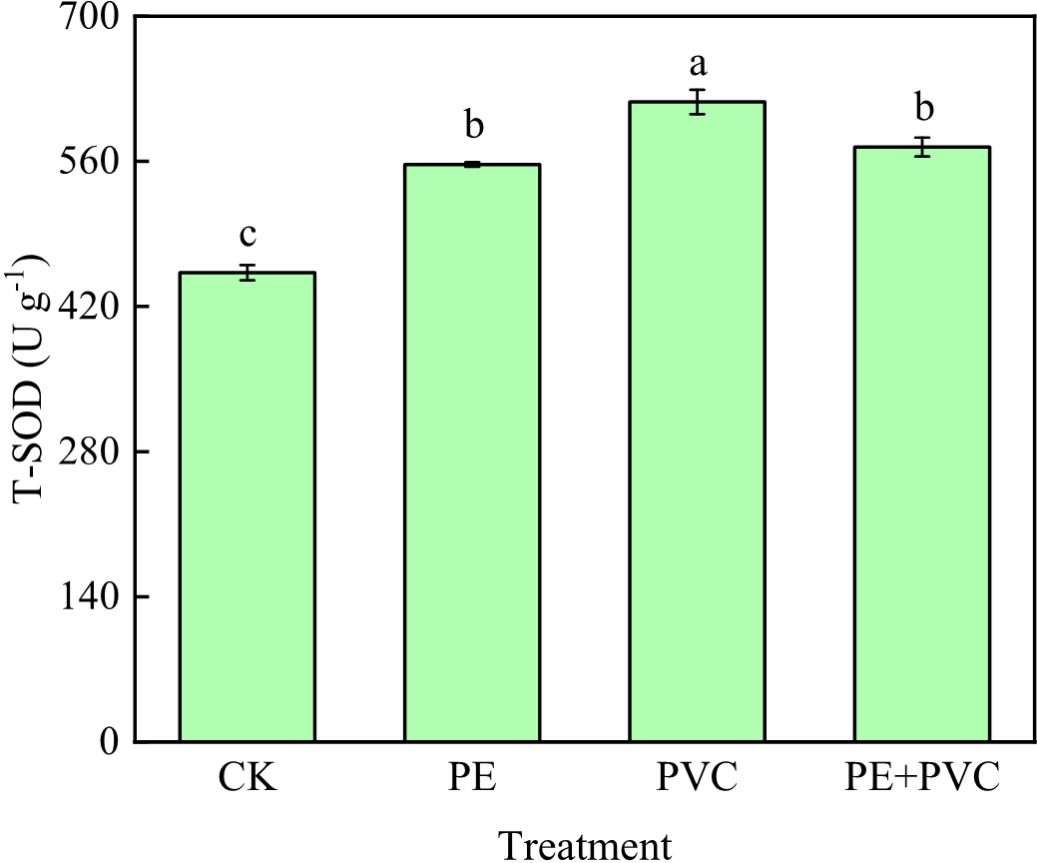
Total-superoxide dismutase (T-SOD) activity of soybean leaves under MPs stress. Different lowercase letters in the graph represent significant differences between treatments (*P*≤0.05).

The changes of T-SOD activity in leaves of soil MPs stressed soybean are shown in **Figure 2**. Different soil MPs treatments significantly increased the activities of T-SOD. The highest value (617.2 U g^-1^) of T-SOD was in the PVC treatments, which was much higher than that in the PE and PE+PVC treatments 60.3 and 43.5 U g^-1^, respectively.

### 3.3 Gas exchange parameters

The effected of PE and PVC, single and in combination, on the leaf photosynthesis of soybean were shown in **Table 1**. PE, PVC, and PE+PVC treatments caused a significant decrease in leaf net photosynthesis rate (*P*_n_), stomatal conductivity (*G*_s_), and transpiration rate (*T*_r_), but had no remarkable influence on intercellular CO_2_ concentration (*C*_i_). Compared to those of the CK treatment, *P*_n_, *G*_s_, and *T*_r_ in PE treatment decreased by 11.9%, 10.5%, and 12.8%, in the PVC treatment decreased by 22.4%, 23.7%, and 30.8%, and in the PE+PVC treatment decreased by 15.7%, 18.4, and 33.3%, respectively.

**Table 1:** Effect of soil MPs stress on gas exchange parameters of soybean.

Treatment	*P*n (μmol m^-2^ s^-1^)	*C*i (ppm)	*T*r (mmol m^-2^ s^-1^)	*G*s (mmol m^-2^ s^-1^)
CK	21.0±0.9 a	303±11 a	7.6±0.1 a	0.39±0.02 a
PE	18.5±0.2 b	309±11 a	6.8±0.2 b	0.34±0.01 b
PVC	16.3±0.1 c	310±5 a	5.8±0.1 d	0.27±0.01c
PE+PVC	17.7±0.5 b	309±10 a	6.2±0.1 c	0.26±0.02 c

The same letters mean no significant differences (P ≤ 0.05) in net photosynthesis rate (P_n_), stomatal conductivity (G_s_), and transpiration rate (T_r_) between different treatments.

### 3.4 Chlorophyll a fluorescence transient

**Figure 3:**
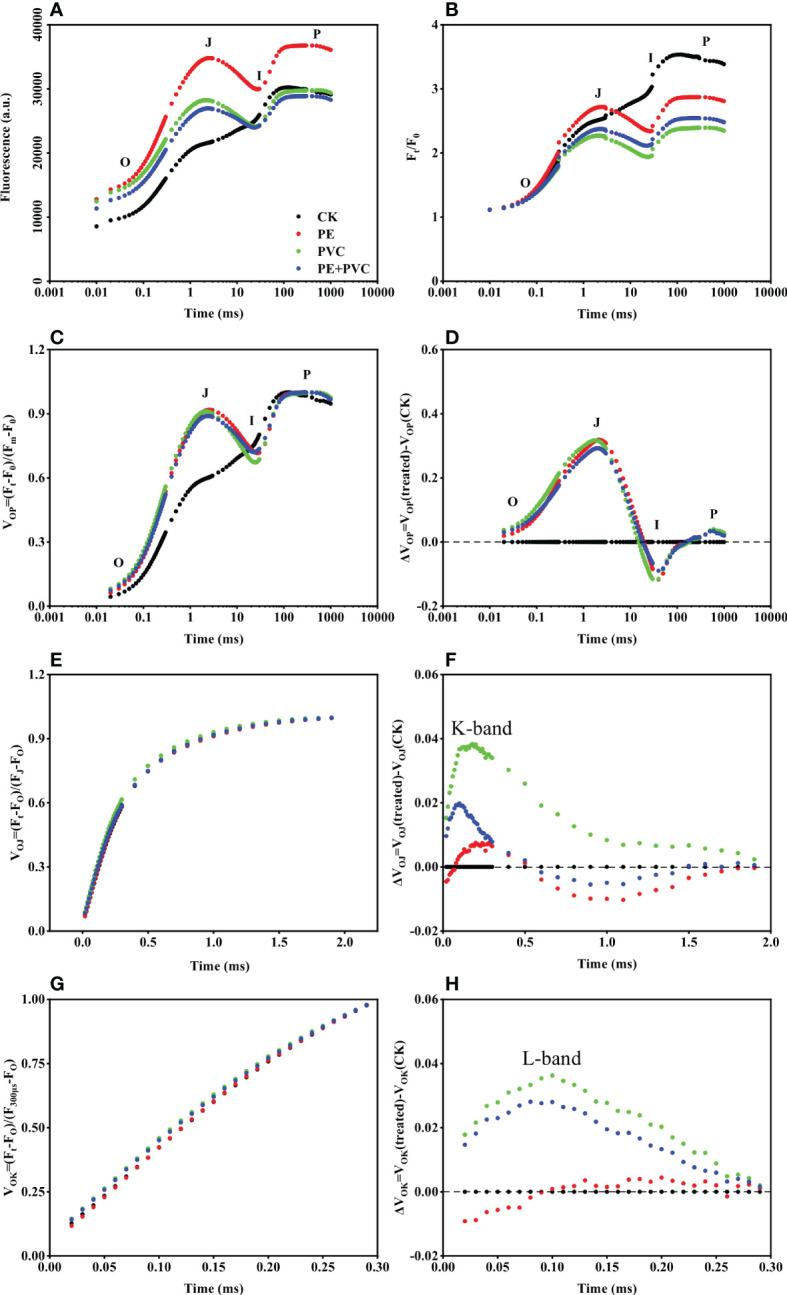
Soybean OJIP fluorescence transients after 30 min of dark adaptation under MPs stress (logarithmic time scale). **(A)** Chl a fluorescence transient curves **(B)** Chl a fluorescence transient normalized data at F_O_(F_t_/F_O_) **(C)** Chl a fluorescence transient normalized at F_O_ and F_m_ (V_OP_) **(D)** variance in Vop between MPs treatment and CK **(E)** Chl a fluorescence transient normalized data normalized between F_O_ and F_J_, phases **(F)** variance in V_OJ_ between MPs treatment and CK **(G)** Chl a fluorescence transient double normalized between F_O_ and F_K_ phases **(H)** variance in V_OK_ between MPS treatment and control.

The Chl fluorescence induction curves from dark adapted leaves suffered different soil MPs stresses were shown in **Figure 3**. All of Chl fluorescence induction curves showed a typical OJIP shape (**Figure 3A**). The O-J phase and J-I phase in the PE, PVC, and PE+PVC treatments were clearly higher than those in the CK treatment. The later part I-P phase in the PE treatment was significant difference with those of the other three treatments. O, J, I and P steps, can be observed in the normalized chlorophyll a (Chl a) fluorescence (F_t_/F_O_) (**Figure 3B**). The addition of PE, PVC, and PE+PVC resulted in a remarkable decrease of the fluorescence at the J-I and I-P phases. The difference between V_OP_ (**Figure 3C**) and CK for the MPs treatment gives ΔV_OP_ (**Figure 3D**), and it can be seen that the MPs treatment has a large difference in the relative rate of change of fluorescence in all three phases of OJI, with peaks and troughs in phases J and I, respectively. Similarly, the K-band and L-band can be clearly seen after making the difference between V_OP_ (**Figure 3E**) and V_OJ_ (**Figure 3G**) proc.

### 3.5 Spider plot of JIP test parameters and energy pipeline models

**Figure 4:**
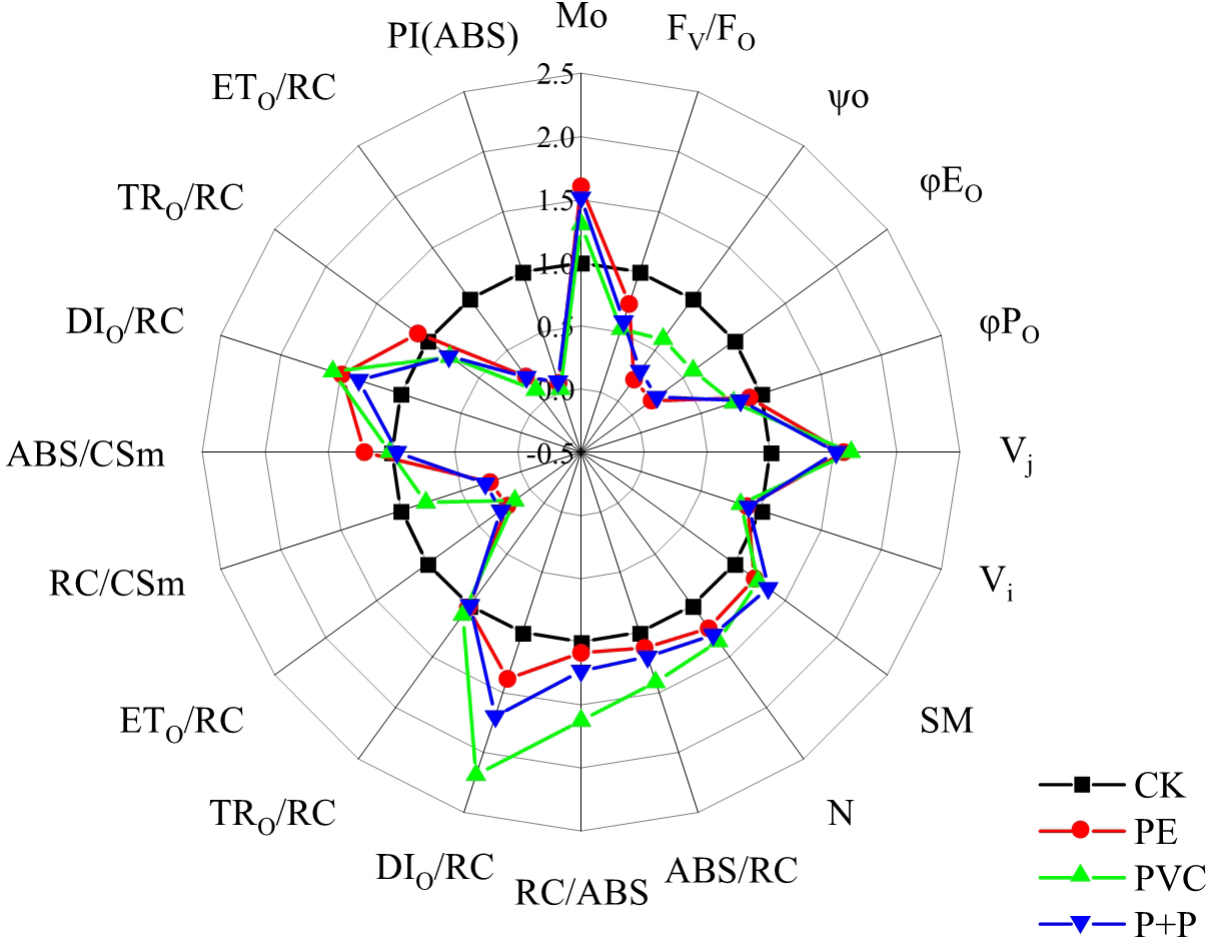
The 'spider plots' of different photosynthetic indicators represent the performance of soybean photosynthetic system II under different MPs stress. All values are proportion of the control treatment (control treatment = 1.0).

The spider plot showing the relative ratio of JIP test parameters of MPs treatments to those of CK treatment (relative to CK) is illustrated in **Figure 4**. By setting the parameters of CK treatment as control, the values of M_O_, V_J_, S_M_, N, ABS/RC, RC/ABS, DI_O_/RC, and DI_O_/CSm of the PE, PVC, and PE+PVC treatments were significant enhanced, while ψ_O_, φE_O_, φP_O_, ETo/RC, RC/CSm, ET_O_/CSm, and PI(ABS) were remarkable downregulated. V_I_, TR_O_/CSm, ABS/CSm and TR_O_/RC in different treatments shows no discernible changes. Among those JIP test parameters, PI (ABS) was most strongly affected by soil MPs stresses. Compared to that of the CK treatment, PI (ABS) of the PE, PVC and PE+PVC treatments decreased by 92.3, 94.7, and 91.0%, respectively.

**Figure 5:**
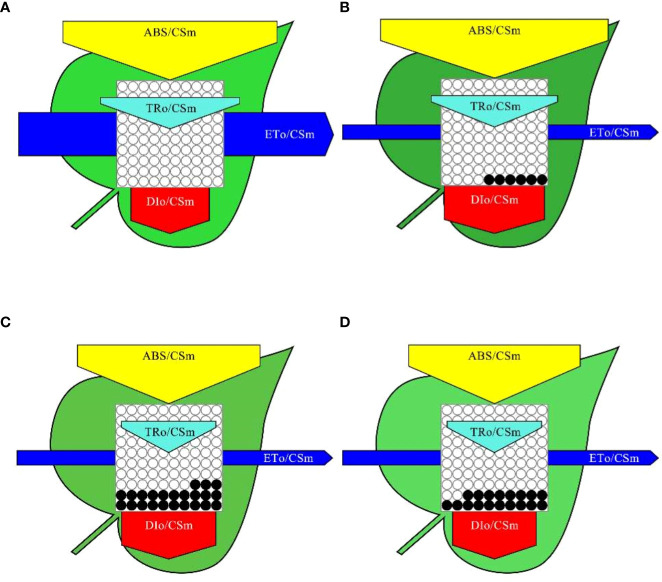
Impact of MPs on soybean energy pipeline models, Calculated according to cross section (CSM) in control **(A)**, PE treatment **(B)**, PVC treatment **(C)**, and PE+PVC treatment **(D)** in soybean PSII. The hollow and solid black circles represent the percentage of active RCs and inactive RCS respectively.

To better visualize the effect of MPs on PSII energy channels, we constructed a model of the photosynthetic system energy pipeline for all the treatments, as shown in **Figure 5**. The width of each arrow in the figure indicates the relative fluxes of energy absorption, capture, transport and dissipation for each excitation cross section in soybean leaf PSII (ABS/CSm, yellow arrow; TR_O_/CSm, cyan arrow; ET_O_/CSm blue arrow; DI_O_/CSm, red arrow). Compared with those of the CK treatment, the relative changes of ABS/CSm, TR_O_/CSm, ET_O_/CSm, and DI_O_/CSm were 9.27%, -4.94%, -68.51%, and 35.56% respectively for the PE treatment, -12.47%, -30.79%, -68.96%, and 21.44% respectively for the PVC treatment, and -19.26%, -33.1%, -60%, and 6.35% respectively for the PE+PVC treatment. Furthermore, we observed that MPs pressure led to increased density of closed reaction centers (RCs) in the PSII cross section (number of black solid circles in **Figure 5**). The maximum of active RCs converted to inactive RCs was 23% for PVC treatment, followed by

PE+PVC treatment (18%) and PE treatment in that order.

### 3.6 Relationship between oxidative damage, Chl content and gas exchange parameters in soybean leaves

Among the five selected parameters (*P*n, Chl a, PI(ABS), MDA, DI_O_/RC), in CK and MPs treatments *P*n, PI(ABS), and Chl a were significantly positively correlated (*P* ≤ 0.05), and CHL and MDA were significantly negatively correlated with correlation coefficients of -0.72, -0.77, -0.98, and -0.72, respectively (*P* ≤ 0.05). There was a significant negative correlation between PI(ABS) and MDA with -0.75 and -0.85 in PVC and PE+PVC treatments, respectively (*P* ≤ 0.05). While two parameters, PI(ABS) and DI_O_/RC, were significantly negatively correlated in PE+PVC treatment (-0.86).

## 4 Discussion

### 4.1 Effect of PE and PVC- MPs on soybean MDA and T-SOD

This study investigated the negative effects of PE-MPs and PVC-MPs in soil, alone or in combination, on photosynthetic performance in leaves of soybean. MPs in soil will cause oxidative stress to plant tissues, which increases the output of plant mitochondrial ROS (Yu et al., 2021; Aliyari Rad et al., 2022). Malondialdehyde (MDA) as a major product of lipid peroxidation, its level reflects the degree of cell membrane damage caused by oxidative stress (Wen et al., 2011). In our present study, compared to CK, all MPs treatments remarkable enhanced the leaf MDA content (**Figure 1A**), it was showed that PE-MPs and PVC-MPs caused lipid peroxidation in soybean, which was manifested as an increase in the MDA content of soybean leaf cells. Furthermore, the MDA contents in PVC-MPs stressed leaves were much higher than that in PE+PVC-MPs treated leaves, indicating the toxic effects of single PVC-MPs on soybean were higher than single PE-MPs and their combinations. In the lettuce study, MPs stress was found to cause an increase in their cellular MDA content (Gao et al., 2019). A complex array of antioxidant systems exists in plants to reduce the damage caused by environmental stresses leading to elevated ROS (Zhang et al., 2021). Among enzymatic antioxidant processes, SOD is the first antioxidant defense against ROS (Zhou et al., 2021). In our study, an obvious increase in SOD activity was found in PE-MPs, PVC-MPs, and PE+PVC-MPs treated plants, PVC MPs treated soybean leaves showed the most significant increase in SOD activity, while PE and PE+PVC treatments showed approximately the same SOD activity (**Figure 2**). Which was compatible with the studies of Jiang et al. (2019). External stress leads to an increase in intracellular ${\text{O}}_{2}^{-}$ concentration, and gene expression of SOD is induced by high ${\text{O}}_{2}^{-}$ concentration expression, leading in turn to increased SOD activity in the plant (Gill and Tuteja, 2010). The greater degree of oxidative damage to soybean by PVC MPs may be due to the fact that PVC MPs are more hydrophilic than PE MPs and thus are absorbed by plants to participate in the plant water cycle process, which in turn leads to greater oxidative damage to soybean leaves treated with PVC MPs (Zhu et al., 2019).

### 4.2 Effect of PE and PVC- MPs on chlorophyll content of soybean leaves

Chloroplasts are not only the primary photosynthetic organisms that perform a serious of photosynthesis processess including light energy absorption, transmission, and conversion to chemical energy but also the major sites of ROS generation under stress conditions (Xu et al., 2006). Previous studies have concluded that environmental stress leads to reduced leaf Chl levels due to lipid peroxidation that causes oxidative damage to chloroplast organs (Bagheri et al., 2019). In our present study, the noticeable decrease of Chl contents in leaves of soybean suffered PE-MPs and PVC-MPs single or composite contamination (**Figure 2A**). PVC-MPs slowly release additives containing toxic substances from the production process in the environment, causing more serious oxidative damage to plants, increased soybean ROS content and T-SOD enzyme activity (Paganos et al., 2022), increased T-SOD enzyme and peroxidase activity decreased photosynthetic gene transcription and chlorophyll reduction (Shahi Khalaf Ansar et al., 2022). We believe that the most severe oxidative damage to PVC-treated soybeans is the main reason for the lowest Chl content in PVC treatment.

### 4.3 Effect of PE and PVC- MPs on gas exchange of soybean leaves

**Figure 6:**
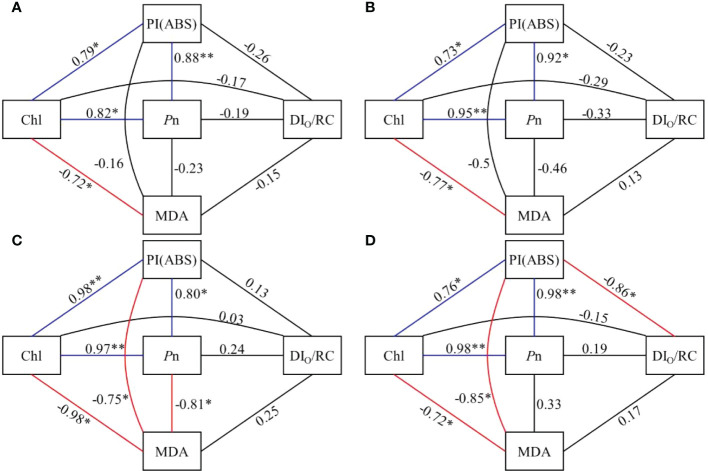
Correlation coefficients between Chl, Pn, PI (ABS), DI_O_/RC and MDA in control **(A)**, PE treatment **(B)**, PVC treatment **(C)**, and PE+PVC treatment **(D)** in soybean (*represents a significant correlation at *P*≤0.05 level, **represents a significant correlation at *P*≤0.01 level). Blue lines indicate a positive correlation and red lines indicate a negative correlation.

Environmental stress is one of the main causes of reduced photosynthetic intensity in plants, and the limitation of photosynthesis will affect the ability of plants to assimilate CO_2_. The accumulation of ROS and reduction of Chl content will ultimately inhibit plants’ photosynthetic efficiency (Song et al., 2020). In our research, PE-MPs and PVC-MPs single or composite contamination significant decreased *P*_n_, *T*_r_ and *G*_s_ in leaves of soybean but had no noticeable effect on *C*_i_, the negative impact caused by PVC treatment was significantly greater than that of the PE+PVC treatment, while the negative effect of the PE+PVC treatment was greater than that of the PE treatment (**Table 1**). PVC-MPs can enrich heavy metals and polycyclic aromatic hydrocarbons (PAHs) in the soil to further stress plants, inhibit photosynthetic pigment synthesis, and reduce the efficiency of light energy conversion (Fatemi et al., 2020). The simultaneous changes in *G*_s_ and *P*_n_ and relative stable values of *C*_i_ suggested that nonstomatal limitation, rather than stomatal limitation, it may be the main reason for the decrease in net photosynthetic rate in soybean leaves subjected to MPs stress (Farquhar et al., 1980). The significant relationship of *P*_n_ with Chl and MDA (**Figure 6**) support the above conclusion. The above results were contrary to the study of Lian et al. (2020), who found that 0.01~ 10 mg L^-1^ polystyrene nanoplastics markedly enhanced *P*_n_, *G*_s_, and *T*_r_ in leaves of wheat seedlings.

### 4.4 Effects of PE and PVC-MPs on chlorophyll a fluorescence transients of soybean leaves

Due to the fragile characteristic of photosystem II (PSII), the structure and function of PSII are the first to be damaged under the influence of external abiotic stresses (Shen et al., 2019). The massive accumulation of ROS in plant cells affects the photosynthetic redox signaling pathway, which manifests as an imbalance in the number of ions on both sides, while the self-repair mechanism of PSII is inhibited, ultimately leading to the inhibition of plant photosynthesis (Song et al., 2020), Moghadam et al (2020) suggested that environmental stress affects photosynthesis and enzyme activity in canola. The effect of PVC-MPs on water uptake by plant roots is higher than that of PE-MPs, so the physiological processes in which plants have water participation are hindered (Kalčíková et al., 2017). To study and assess the functional state of PSII under different MPs stress conditions, measurements of Chl a fluorescence kinetics as a powerful tool in reflecting the reduction of photosynthetic electron transport chain were performed in the present study (Liu et al., 2017). The fluorescence induction curves of PE and PVC MPs stressed soybeans showed a multiphase continuous upward trend (O-J-I-P) under the influence of PE and PVC-MPs (**Figure 3**). The results showed that PE-MPs and PVC-MPs single or composite contamination had multiple negative effects on soybean PSII. PE-MPs and PVC-MPs single or composite stress led to an increase in soybean F_O_, but the difference between treatments was not significant. PVC-MPs and PE+PVC-MPs did not increase soybean Fm, and only PE-MPs led to an increase in soybean Fm. The fluorescence transients of soybean under MPs stress were higher than those of CK until stage I, but the fluorescence transients of soybean after stage I of PVC and PE+PVC treatments were similar to those of CK (**Figure 3A**). We found that exposure of soybean to PE and PVC MPs leads to an increase in F_O_ and FM, which may be due to two reasons, one being the effect of PE and PVC- MPs on causing damage to the photosynthetic structure of the plant (e.g., reduced chlorophyll content) which in turn affects the lack of energy transfer from the photosynthetic antenna to the active RCs, leading to an increase in F_O_ (Goussi et al., 2018), and the other being that environmental stress increases the shift from active to inactive RCs, which in turn inhibits the process of Q_A_ reduction, ultimately leading to an increase in F_O_ (Lu et al., 2001). However, the increase in F_O_ instead led to a decrease in the relative increase in soybean fluorescence transients, which was greatest in the PVC treatment (**Figure 3B**), which is consistent with the findings of Goussi et al. (2018).

ΔV_OP_ can help us to clearly see the difference in fluorescence transient changes of soybean among treatments by offsetting the difference in Fm and F_O_ among treatments to obtain the relative fluorescence change V_OP_, and then compare that with the difference in V_OP_ of CK treatment (Murakami and Ae, 2009). The variation of ΔV_OP_ was approximately the same as the three MPs treatments in our study. It can be clearly seen that there is a decrease between J and I phases and the lowest negative peak is observed in I phase (**Figure 3D**), which indicates, to some extent, the decrease in the efficiency of the J-I phase electron transport link (Stirbet and Govindjee, 2011), I-P phase electron transport link (Redondo-Gómez et al., 2010). The occurrence of L-band and K-band of fluorescence transients can respond to the extent to which the photochemical efficiency of plants is affected by environmental stresses (Strasser and Stirbet, 2001). Oukarroum and Stirbet (2011) suggested that the reduced energy transfer between the chloroplast photosynthetic antenna and PSII RCs leads to the appearance of L-band on the one hand, and the imbalance in the number of electrons on the donor and acceptor side of PSII leads to the appearance of K-band on the other. Under the influence of PE and PVC MPs, both L-band (**Figure 3H**) and K-band (**Figure 3F**) were present in our study, and the PVC treatment produced higher L- and K-bands than the PE and PE+PVC treatments. Compared to PE, PVC-MPs may leach the contained chlorides and therefore be more toxic to plants (Fuller and Gautam, 2016). These results indicate that soybean exposure to MPs severely limits the flow of electrons from OEC to RCs and from PSI units to PSII units (K-band), while the connectivity of energy transfer between PSII units is also limited (L-band), PVC-MPs caused the most significant impact on both of these limitations. In the study by Wang et al. (2021) on chlorella vulgaris stressed by PVC-MPs, in the molecular structure, vinyl chloride has one more chlorine atom than ethylene. PVC can enrich the soil with heavy metal elements or pesticide residues, resulting in PVC-MPs being more biotoxic than PE-MPs, resulting in more severe oxidative damage to soybeans and affecting photosynthesis in soybeans. (Chen et al., 2020; Song et al., 2020), becoming the cause of the reduced energy transfer between PSII RCs and the imbalance in the number of electrons on the donor and acceptor sides of PSII.

### 4.5 Effect of PE and PVC-MPs on fluorescence parameters of soybean leaves

Analysis of multiple fluorescence parameters provides further insight into the extent of damage caused by PE and PVC-MPs to different parts of the soybean photosynthetic system. Numerous studies have shown that the PSII donor-side hydrolysis complex efficiency (F_V_/F_O_) is most susceptible to environmental stresses. The reduction of hydrolytic complex efficiency inhibits photosynthetic electron transport (Pereira et al., 2000). All three MPs treatments had different degrees of reduction in F_V_/Fo, and the PVC treatment had the lowest Fv/Fo, indicating that the PVC produced the greatest environmental stress. The toxicity of PVC MPs in soil was also concluded to be higher than that of PE-MPs in the study by Zhu et al. (2019). There is a viable way for MPs to transfer their own chemical contaminants and additives into plant tissues, posing a potential risk, and in addition PVC-MPs contain plasticizers and oligomers that can inhibit seed germination rates, affect normal plant physiological processes, and even cause programmed plant cell or organelle death (Wathsala et al., 2018; Campanale et al., 2020). Manaa (2021) suggested that an increase in V_J_ is a typical feature of plants subjected to environmental stress. In our study, all three MPs treatments showed the same degree of increase in VJ, suggesting that electron transfer to PSII reaction centers is restricted under the influence of MPs (Mehta et al., 2010). φPO (FV/Fm) can be used to determine the PSII primary optical maximum quantum yield, and its decrease indicates that redox is inhibited after Q_A_ flipping (Schansker et al., 2005). φPO decreased less in our study, which indicates that this index is less affected by MPs. PI (ABS) is a performance indicator of photosynthesis, and its value magnitude reflects whether plant photosynthesis is affected or not (Lian et al., 2020). PI(ABS) is also highly susceptible to environmental stress, so it can make an evaluation of the plant health under environmental stress (Li et al., 2020). In this study, PI(ABS) showed a significant decrease under the influence of MPs, while there was a significant negative correlation between PI(ABS) and leaf MDA content in PVC and PE+PVC treatments (**Figure 6**), indicating that PVC-MPs stress leads to oxidative stress in soybean, and an increase in the degree of oxidative stress leads to a decrease in soybean photosynthetic performance indexes.

ABS/RC can be considered as the effective antenna size of active RCs, and the antenna size of active RCs increases under environmental stress, and this effect is often caused by a decrease in the ratio of active RCs (Mehta et al., 2010). PE-MPs and PVC-MPs single or composite contamination had an effect on both the reduction in the ratio of active RCs and the increment in the effective antenna size of active RCs, with the PVC treatment producing the largest effect, it has been confirmed in this experiment. DI_O_/RC is a self-protective mechanism of plants that reduces excess absorbed heat energy by means of heat dissipation thus protecting plants from damage, but the increase in DI_O_/RC leads to the enhancement in the number of inactive RCs (Kalaji et al., 2011). In our study, we found that DI_O_/RC and ET_O_/RC increased and decreased, respectively, under the influence of PVC-MPs, but TR_O_/RC was not affected by PVC-MPs in the environment. This suggests that the energy capture by active RCs does not change under the influence of PE and PVC-MPs, but the reduction in electron transport efficiency and reaction center activity leads to a self-protection mechanism in plants, resulting in an increase in thermal energy dissipated by RCs, which in turn decreases the amount of active RCs and thus has an impact on the net photosynthetic rate. The difference is that the percentage reduction in energy fluxes for absorption flux (ABS/CSm), trapped energy flux (TRo/CSm) and dissipated energy flux (DIo/CSm) per excited cross section (**Figure 5**) is significantly smaller than the percentage reduction in electron transport fluxes per excited cross section (ETo/CSm), suggesting that soybean electron transport fluxes are more susceptible to reduction under the stress of PE-MPs and PVC-MPs single or composite contamination and that this change also leads to an increase in the proportion of active RCs to inactive The proportion of RC conversion increases.

## 5 Conclusions

We carried out a study on the effect of photosynthesis in soybean leaves at flowering stage under the stress of PE-MPs and PVC-MPs alone as well as under their combined stress. It was found that PE-MPs and PVC-MPs, alone or co-existence, all posed serious oxidative damage on soybean plants, resulting in increase of lipid peroxidation and T-SOD activity and decrease of Chl content, the addition of PVC-MPs in soils caused more oxidative stress to soybean plants than PE-MPs and PE+PVC-MPs, suggested that the PVC-MPs had a higher toxicity effect than PE-MPs.

The changes in gas exchange parameters (*P*_n_, *G*_s_, and *C*_i_) under MPs stress conditions, suggesting that stomatal limiting factors are responsible for MPs stress affecting photosynthesis in soybean, PVC-MPs single contamination had the greatest effect on photosynthetic gas exchange parameters in soybean.

Based on the analysis of OJIP fluorescence kinetics and the construction of leaf energy fluxes phenomenological model, soybean photosynthetic antennae show reduced and unbalanced energy connectivity with PSII RCs as well as electrons on both PSII donor and acceptor sides, while the number of soybeans PSII RCs is reduced under the influence of PE and PVC-MPs, which in turn leads to inhibition of Q_A_ redox as well as increased energy dissipation.

The effects of single contamination by PVC-MPs on oxidative stress, gas exchange, and PSII function and structure in soybean were greater than those of single contamination by PE-MPs and co-contamination by PE and PVC-MPs, and the above results indicate that there is no enhancement effect of co-contamination by PE and PVC-MPs on oxidative stress and photosynthesis in soybean.

## Data availability statement

The original contributions presented in the study are included in the article/**Supplementary Material**. Further inquiries can be directed to the corresponding authors.

## Author contributions

HL and FS conceived and designed the experiments. HL and FS conducted laboratory analyses. KZ and QL analyzed the data and wrote results. HL, GN, and JZ wrote the manuscript (Introduction and Discussion). All authors provided editorial advice and revised manuscript.
