# Supplementary material for: Single and composite damage mechanisms of soil polyethylene/polyvinyl chloride microplastics to the photosynthetic performance of soybean (Glycine max [L.] merr.)
Source: Front Plant Sci. 2023 Jan 18;13:1100291. doi: 10.3389/fpls.2022.1100291 (PMC9889878; doi:10.3389/fpls.2022.1100291)
Supplement: Supplementary file 1 [file Table_1.docx]

**Table S1. Explanations of selected fluorescence parameters used in the present study.**

| Fluorescence parameters | Description |
| --- | --- |
| F_O_ | Minimal(t=μs) fluorescence when all the reaction centers are open |
| F_m_ | Maximal recorded fluorescence intensity |
| F_V_ | Variable chlorophyll fluorescence（F_m_-F_v_） |
| F_J_≡ F_2ms_ | Fluorescence intensity at the J-step (2 ms) |
| F_I_ ≡ F_30ms_ | Fluorescence intensity at the I-step (30 ms) |
| M_O_ = (∆V / ∆t)_O_ = 4(F_300μs_ − F_O_) / (F_m_ − F_O_) | Approximated initial slope (in ms^−1^) of induction curve Vt (for F_O_ = F_20μs_) |
| ψ_O_= ET_O_ / TR_O_ = (1 − V_J_) | Probability that a trapped exciton moves an electron further than Q_A_^−^ |
| φE_O_ = ET_O_ / ABS = [1 − (F_O_/F_m_)] ψ_o_ | Probability that an absorbed photon moves an electron further than Q_A_^−^ |
| φP_O_ = TR_O_ / ABS = F_v_ / F_m_ = [1 − (F_O_ / F_m_)] | Maximum quantum yield of primary photochemistry (at t = 0) |
| V_J_ = (F_J_ − F_O_) / (F_m_ − F_O_) | Relative variable fluorescence at the J-step |
| V_I_= (F_I_ − F_O_) / (F_m_ − F_O_) | Relative variable fluorescence at the I-step |
| S_M_ = Area / F_V_ | Normalized area (reflecting multiple turnover Q_A_ reduction events and representing energy necessary for the closure of all reaction centers |
| N = S_M_ M_O_ (1 / V_J_) | Number of Q_A_ redox turn over until Fm is reached |
| ABS / RC = M_O_ (1 / V_J_) (1 /φP_o_) | Absorption flux (for PSII antenna chlorophylls) per reaction center (RC) |
| DI_O_ / RC = (ABS / RC) − (TR_O_ / RC) | Dissipated energy flux per reaction center RC (at t = 0) |
| TR_O_ / RC = M_O_ (1 / V_J_) | Trapped energy flux (leading to Q_A_ reduction) per reaction center RC |
| ET_O_ / RC = M_O_ (1 / V_J_) ψ_o_ | Electron transport flux (further than Q_A_^−^) per PSII RC (at t = 0) |
| RC / CSm = ψ_o_ (V_J_ / M_O_) (ABS / CS_m_) | Density of reaction centers per excited cross-section (at t = t_Fm_) |
| ABS / CSm = F_m_ (at t = t_Fm_) | Absorption flux per excited cross section, approximated by F_m_ |
| DI_O_ / CSm = (ABS / CS_m_) − (TR_O_ / CS_m_) (at t = tF_m_) | Dissipated energy flux per excited cross section, approximated by F_m_ |
| TR_O_ / CS_m_ = φP_o_ (ABS / CS_m_) (at t = t_Fm_) | Trapped energy flux per excited cross section, approximated by F_m_ |
| ET_O_ / CS_m_ = φE_o_ (ABS / CS_m_) (at t = t_Fm_) | Electron transport flux per excited cross section, approximated by Fm |
| PI(ABS) = (ABS/RC) (φP_o_ /(1-φP_o_)) (ѱo/ (1- ѱo)) | Performance index on absorption basis |
| PI(CS_m_) = (RC / CS_m_) (φP_o_ / (1 − φP_o_)) − (ψ_o_ / (1 − ψ_o_)) | Performance index on cross section basis |
